# Standardization of PCR-RFLP analysis of nsSNP rs1468384 of NPC1L1 gene

**DOI:** 10.4103/0971-6866.44999

**Published:** 2008

**Authors:** Praveen P. Balgir, Divya Khanna, Gurlovleen Kaur

**Affiliations:** Department of Biotechnology, Punjabi University, Patiala-147 002, India

**Keywords:** Influx transporter, M510I, NPC1L1, single nucleotide polymorphisms

## Abstract

Niemann-Pick C1-like 1 (NPC1L1) protein, a newly identified sterol influx transporter, located at the apical membrane of the enterocyte, which may actively facilitate the uptake of cholesterol by promoting the passage of sterols across the brush border membrane of the enterocyte. It effects intestinal cholesterol absorption and intracellular transport and as such is an integral part of complex process of cholesterol homeostasis. The study of population data for the distribution of these single nucleotide polymorphisms (SNP) of NPC1L1 has lead to the identification of six non-synonymous single nucleotide polymorphisms (nsSNP). The in vitro analysis using the software MuPro and StructureSNP shows that nsSNP M510I (rs1468384), which involves A→G base pair change leads to decrease in the stability of the protein. A reproducible and a cost-effective PCR-RFLP based assay was developed to screen for the SNP among population data. This SNP has been studied in Caucasian, Asian, and African American populations. Till date, no data is available on Indian population. The distribution of M510I NPC1L1 genotype was estimated in the North Western Indian Population as a test case. The allele distribution in Indian Population differs significantly from that of other populations. The methodology thus proved to be robust enough to bring out these differences.

## Introduction

Niemann-Pick C1-like 1 (NPC1L1) protein, a newly identified sterol influx transporter, located at the apical membrane of the enterocyte, which may actively facilitate the uptake of cholesterol by promoting the passage of sterols across the brush border membrane of the enterocyte.[1,2] The protein has been characterized by the presence of a signal peptide, 13 putative transmembrane regions, a conserved NPC1 domain and a sterol sensing domain (SSD). Although expressed within several tissues in the body, the expression is predominant in liver and small intestine.[3–5] In rodents, NPC1L1 is highly expressed on the surface of jejunal absorptive cells whereas in humans the expression is more in the hepatoma cells in the liver.[6] The protein plays a key role in cholesterol uptake and intracellular cholesterol trafficking from the plasma membrane to the endoplasmic reticulum.[7] A fact further strengthened by the observation of Gracio-Calvo *et al*.[8] that the protein was the direct molecular target of ezetimibe, a drug that inhibits cholesterol absorption. Recently, Temel *et al*.[9] have suggested the presence of NPC1L1 on the canalicular membrane of hepatocytes which may modulate biliary cholesterol excretion. Thus, this protein is actively involved in the cholesterol homeostasis pathway.[10,11]

Single nucleotide polymorphisms (SNPs), together with copy number variation, are the primary source of variability in the human genome. As amino acid substitutions currently account for approximately half of the known gene lesions responsible for human inherited disease, study of non-synonymous single nucleotide polymorphisms (nsSNPs) are important in delineating the etiology of many such disorders.[12,13] These SNPs may lead to changes in protein confirmation and may be associated with altered response to drug treatment, susceptibility to disease, and other phenotypic variations.[14] The study of population data for the distribution of NPC1L1 has lead to the identification of six nsSNPs. Among the 6 identified nsSNPs, M510I (rs1468384) shows decrease in the stability of the protein as analyzed in silico by MuPro[15] and StructureSNP[16] softwares. The M510I polymorphism is the result of a nucleotide change G to A at position 2993 of the cDNA sequence in exon 2, and it results in the substitution of isoleucine for methionine at amino acid 510 of the NPC1L1 protein. The SNP has already been studied in Caucasian, Asian, and African American populations by sequencing as given in NCBI database. Till date, no data is available on Indian population. Thus, a reproducible and cost-effective PCR-RFLP based assay was developed to study the distribution of this SNP as well as its frequency distribution comparison with other world populations.

## Materials and Methods

High molecular weight genomic DNA was extracted from 3.0 ml of the blood samples collected from 150 normal healthy individuals in the age group 20-50 years with informed consent. The DNA was isolated by methodology as given by Lahiri *et al*.[17]

### Primer designing

The primers for the PCR were designed by using the software GENE RUNNER Version 3.05. The selected primers are listed in the Table 1.

**Table 1:** The primer pair selected by GENE RUNNER version 3.05

S.No.	Primer	Tm	%GC	Blast

	Sequence	Y(°C)		(No. of Hits)
1.	5’AAGCACAGCGCAACATCTCC3’	55.2	55.0	3
	ACAAACCTGCAGCCATGAGC	55.0	55.0	12
2.^**^	5’TATGGTCGCCCGAAGCACAG3’	58.2	60.0	1
	GATGGCCACGCACAAACCTG	58.5	60.0	3
3.	5’CAGGTATGGTCGCCCGAAG	55.3	63.2	1
	CACAAACCTGCAGCCATGAC	53.7	55.0	10
4.	5’CGAAGCACAGCGCAACATCTC	58.2	57.1	4
	GATGGCCACGCACAAACCTG	58.5	60	3

### PCR reaction optimization

The PCR reaction was optimized for 200 ng of DNA. The primer pair selected was 5’ T A T G G T C G C C C G A A G C A C A G 3’ and 3’GATGGCCACGCACAAACCTG5’ were designed using GENERUNNER version 3.05 (Hastings Software Inc. Hastings, NY, USA (http://www.generunner.com). A 25 µL PCR mixture was optimized containing 1.5 mM MgC1_2_, 0.4 μM of each primer (Imperial Genetics, USA), 200 μM of each deoxynucleotide triphosphate (Fermentas, USA), 10% Glycerol (Sigma, USA), 1.0 U of Taq polymerase (Intron Technologies, Germany), and buffer concentration of 50 mM KC1 and 10 mM Tris- HCl, pH 8.4. A two-step PCR cycles were optimized, with first step with initial denaturation at 95°C followed by 30 cycles of denaturation at 95°C for 1 min, annealing at 61.5°C for 1 min and extension at 72°C for 1.30 min. This is followed by a final extension (72°C, 5 min) and a 4°C hold. The annealing temperature (Ta) was optimized at 61.5°C. The temperature was calculated using the formula: Ta = 0.3 * Tm primer + 0.7 * Tm product - 14.9, where Tm product = 83°C, and it was Ta = 60.5 °C (±1 °C).The PCR product of size 437 bp was obtained and stored at 4 °C.

### RFLP analysis

Five units of BccI (New Englands Biolab) was added to 15 μL of PCR product and incubated overnight at 37°C. Restriction Enzyme BccI with the following recognition site was selected from New England Biolab website http.//www.neb.com/. All of the digested products were electrophorezed on 10% Polyacrylamide Gel Electrophoresis. The gel was ran at 150V for 3 h. The gel was visualized by Silver Staining.[18]

5’. . . C C A T C (N)_4_ ▼. . . 3’

3’. . . G G T A G (N)_5_ ▼. . . 5’

### PCR product purification and sequencing

**Figure 1:**
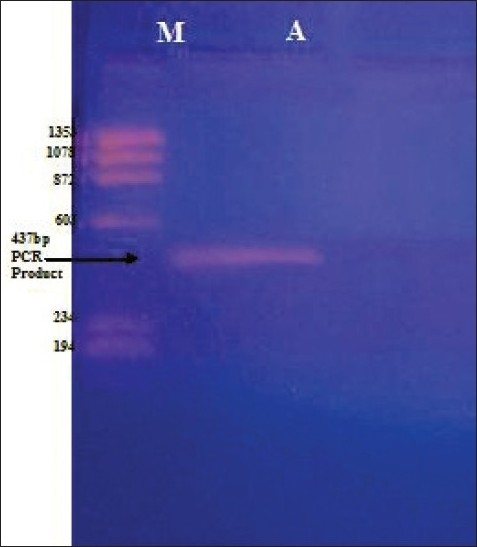
The 1.5% agarose showing purified PCR product of size 437 bp with ΦX174 DNA/ HaeIII digest as marker

The PCR product was purified by Na acetate method.[19] To the PCR product added 1/10^th^ volume of Na-Acetate (3M) followed by twice the volumes of 100% absolute chilled ethanol. The samples were then incubated at -20°C for 1 h. After the incubation period, the sample were centrifuged for 15 min at 10000 RPM at 4°C. Discarded the supernatant and to the pellet added 200 μl of cold 70% Ethanol and re-centrifuged it for 5 min at 4°C. Discarded the supernatant and dissolved the pellet in Tris-EDTA buffer. The purified sample was checked on 1.5% agarose gel as shown in Figure 1. The sample was ready for sequencing. The purified sample was submitted to Banglore Genei at Bangalore, India for sequencing.

## Results and Discussion

An efficient PCR reaction not only generates product of requisite size but also should utilize the primers completely. Thus, a minimal difference in their melting temperatures (Tm) is favorable[20] and in the case of all primer pairs selected as per Table 1 the Tm difference is of 1°C. Both primers and target sequence affect this efficiency.

At room temperatures nucleic acids fold into conformations (secondary structures) which have high negative free energy. The stability of these template secondary structures depends largely on their free energy and melting temperatures and is extremely important for designing primers.[21–23] Keeping these guidelines in mind, the four primer pairs were selected. The primer pair finally selected, had least number of secondary structures, with no hair pin loop, no bulge loops and just 1 internal loop in sense strand at the temperature range during PCR. Further, the 2 dimers in sense strand and 1 in antisense strand were observed to be formed at temperatures far below the experimental temperature range. Moreover the primers so designed were unique i.e., they targeted and amplified only the specific gene in the genomic DNA as given by the Blast[24] [Table 1]. The second primer pair gave minimum and unique hits and was thus selected.

**Figure 2:**
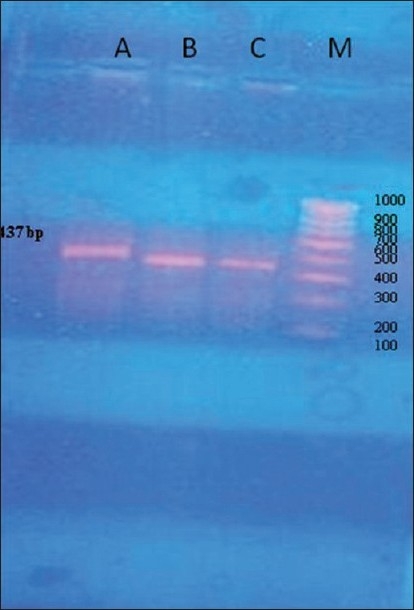
A 1.5% agarose gel depicting NPC1L1 PCR product of size 437 bp at different annealing temperatures (Lane A- 61.5°C, Lane B- 60.5°, Lane C- 59.5°C and Lane M -Marker)

The PCR reaction was set at three different annealing temperatures i.e., 59.5°C, 60.5°C, and 61.5°C. The results are shown in the Figure 2. The annealing temperature of 61.5°C proved to be the most stringent, giving optimum results at which no spurious amplification were observed in the PCR products as compared to other temperatures [Figure 2].

**Figure 3:**
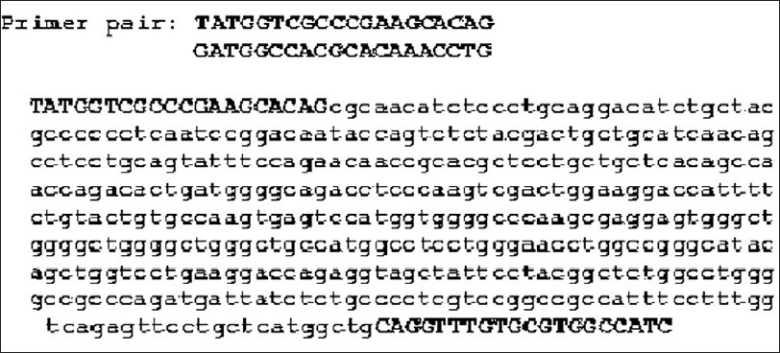
Sequence of PCR product amplified as obtained from Bangalore Genei and viewed in CHROMAS

The optimization of amount of Taq polymerase and number of PCR cycles was then carried out. The amount of Taq polymerase was tested in the range 0.25 to 2 U per reaction. As little as 0.5 U enzymes could be used without a decrease in the yield of PCR product (data not shown). PCR was performed for 25, 30, and 35, cycles, including the initial cycle. The PCR products could be detected after 25 cycles, maximal PCR product was obtained with 30 cycles of PCR amplification without the production of nonspecific amplification and complete utilization of primers (data not shown). The product was purified and sequenced to confirm the region amplified. The Figure 3 depicts the sequence of the PCR product as obtained from Bangalore Genei and viewed in CHROMAS.

**Figure 4:**
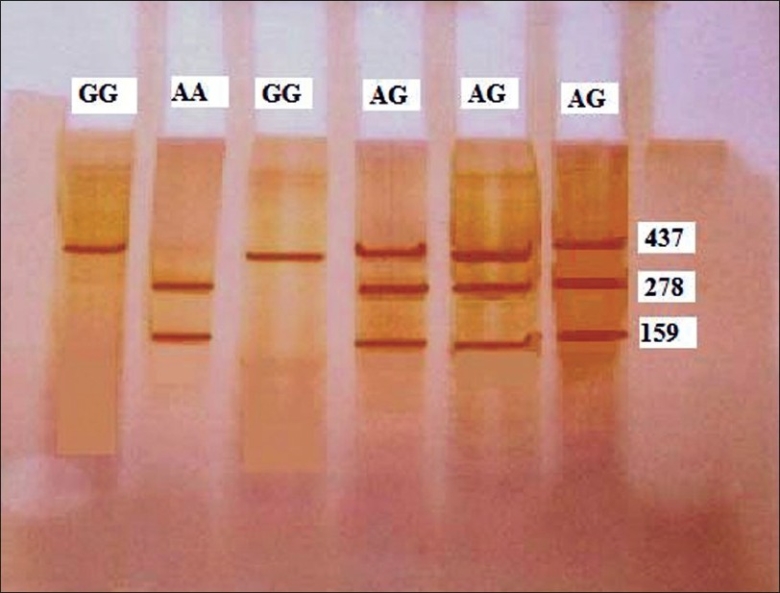
Determination of the M510I genotype by PCR amplification and restriction analysis

The amplified product was subjected to digestion by Bcc1 restriction enzyme. After digestion of the 437 bp fragment obtained by PCR, the three possible genotypes were distinguishable: homozygous GG (437 bp), heterozygous GA (437, 278 and 159 bp), and homozygous AA (278 and 159 bp). The Table 2 and the gel picture Figure 4 below depicts the band pattern for different genotypes.

**Table 2:** The band pattern obtained after digestion with Bcc1 enzyme

Allotypes	AG	AA	GG
Band pattern	437	–	437
	278	278	–
	159	159	–

The genotype frequency distribution for the M510I polymorphism is shown in Table 3. The difference in allele frequency distribution between different population groups was observed. The “G” allele present in Indian Population showed the least frequency of 0.46 (95% CI) and Caucasian Population samples the maximum at 1.000. For “A” allele it is 0.54 (95% CI) in Indian Population [Table 4]. The data was then analyzed for χ^2^ value to study difference in allele distribution amongst the Indian population and other populations of the world studied so far [Table 5]. Statistically, highly significant differences were observed on comparison with these populations. Even the population labeled as Asian on closer scrutiny was found to be a mixed group constituted of populations of mongoloid origin like the Chinese, Malaysians, etc. This group also showed the allele distribution to be highly significantly different from the North Western Indian Population under study.

**Table 3:** NPC1L1 genotype distribution amongst North Western Indian population

S.No.	Genotype	Genotype distribution in normal population	
		Frequency	%age distribution
1.	AA	49	32.6
2.	GG	36	24
3.	AG	65	43.3

		Total	150

**Table 4:** NPC1L1 allele frequency distribution amongst different populations

Sample group	Total no. of sample (2n)	Frequency of G	Frequency of A
African American	78	0.990	0.010
Caucasian	80	1.000	0.00
Asian	60	1.000	0.00
CEPH	184	1.000	0.00
Japanese	86	0.980	0.020
Indian	300	0.460	0.540
(Present Study)			

**Table 5:** Comparison between North Western Indian population and other populations of the world

Sample group	χ^2^	Probability	Significance
African American/ Indian	70.45	<0.0001	HS
Caucasian/Indian	73.9	<0.0001	HS
Asian / Indian	3.9	<0.0001	HS
CEPH/Indian	73.9	<0.0001	HS
Japanese/Indian	67.6	<0.0001	HS

HS - Highly Significant P≤0.05

The above analysis makes it very clear that the PCR-RFLP methodology optimized in current study is robust enough allowing screening of populations for NPC1L1 SNP rs1468384. The earlier studies using sequencing are not only expensive but also not amenable to high throughput population screening.

## Conclusion

The PCR-RFLP technique described here is a proficient NPC1L1 genotyping assay starting with 50 ng genomic DNA extracted from 0.3 mL of blood that makes it cost effective as well as time and labor saving. The PCR product enhancement and specificity were improved by using 10% glycerol. The use of less Taq polymerase, single-step PCR cycling, and polyacrylamide gel electrophoresis not only improved efficiency but also lowered the cost per test. The protocol is thus useful for clinical diagnostic laboratory as well as a research laboratory performing population screening. The importance of study of this SNP lies in the fact that it is predicted to lead to localized structural instability that may have functional implications in disorders involving cholesterol influx, drug binding and susceptibility to complex disorders.
